# Personality traits in companion dogs—Results from the VIDOPET

**DOI:** 10.1371/journal.pone.0195448

**Published:** 2018-04-10

**Authors:** Borbála Turcsán, Lisa Wallis, Zsófia Virányi, Friederike Range, Corsin A. Müller, Ludwig Huber, Stefanie Riemer

**Affiliations:** 1 Clever Dog Lab, Comparative Cognition, Messerli Research Institute, University of Veterinary Medicine Vienna, Medical University of Vienna and University of Vienna, Vienna, Austria; 2 Department of Ethology, Eötvös Loránd University, Budapest, Hungary; 3 Research Centre for Natural Sciences, Institute of Cognitive Neuroscience and Psychology, Hungarian Academy of Sciences, Budapest, Hungary; 4 Division of Animal Welfare, Vetsuisse Faculty, University of Bern, Bern, Switzerland; University of Pisa, ITALY

## Abstract

Individual behavioural differences in pet dogs are of great interest from a basic and applied research perspective. Most existing dog personality tests have specific (practical) goals in mind and so focused only on a limited aspect of dogs’ personality, such as identifying problematic (aggressive or fearful) behaviours, assessing suitability as working dogs, or improving the results of adoption. Here we aimed to create a comprehensive test of personality in pet dogs that goes beyond traditional practical evaluations by exposing pet dogs to a range of situations they might encounter in everyday life. The Vienna Dog Personality Test (VIDOPET) consists of 15 subtests and was performed on 217 pet dogs. A two-step data reduction procedure (principal component analysis on each subtest followed by an exploratory factor analysis on the subtest components) yielded five factors: Sociability-obedience, Activity-independence, Novelty seeking, Problem orientation, and Frustration tolerance. A comprehensive evaluation of reliability and validity measures demonstrated excellent inter- and intra-observer reliability and adequate internal consistency of all factors. Moreover the test showed good temporal consistency when re-testing a subsample of dogs after an average of 3.8 years—a considerably longer test-retest interval than assessed for any other dog personality test, to our knowledge. The construct validity of the test was investigated by analysing the correlations between the results of video coding and video rating methods and the owners’ assessment via a dog personality questionnaire. The results demonstrated good convergent as well as discriminant validity. To conclude, the VIDOPET is not only a highly reliable and valid tool for measuring dog personality, but also the first test to show consistent behavioural traits related to problem solving ability and frustration tolerance in pet dogs.

## Introduction

Domestic dogs play various roles in human society. Highly trained dogs serve as guide dogs for the blind [1], assistance dogs for people with hearing or mobility impairments [2] or autism spectrum disorders [3], therapy dogs [4], military dogs [5], police dogs [6], and search and rescue dogs [7]. However, the most common role of dogs in human hands today is to provide companionship to their human caretakers [8,9].

In an Australian questionnaire study, behavioural characteristics such as a calm/compliant demeanour, high sociability, a lack of aggressiveness, and a high energy level were considered as important by people describing the ‘ideal companion dog’ [10]. If we can test or predict such individual predispositions, it would be highly valuable for matching puppies or adult dogs with the right families and selecting suitable dogs for particular jobs. Thus, the topic of individual behavioural differences—or personality—in domestic dogs has received a lot of scientific interest in recent decades, with publications having approximately doubled in the last ten years (reviewed by [11]).

While some studies have attempted to identify personality × environment associations [1,12,13], heritability and the genetic background of personality traits (e.g. [14–16]), as well as behavioural development in dogs [17–19], the majority of existing tools to measure personality—or rather a few particular aspects of it—come from an applied background. Published tests of dog personality (also referred to as ‘temperament tests’ in many publications) largely fall into three categories: puppy tests designed to predict adult behavioural tendencies or suitability for particular jobs from a young age (e.g. [6,15,17,20–24], tests for assessing individuals’ propensity to react aggressively (e.g. [25]) or identify other potentially problematic behaviours [26–29], and tests for working dogs [1,5,30–32].

Accordingly, tests designed for different purposes try to answer very different questions. It is thus not surprising that methods subsumed as “personality tests” or “temperament tests” differ widely depending on the experiementer’s goals. Different ways of measuring personality have been employed with test batteries and questionnaires being used most often (reviewed in [11]). The advantage of using a test battery lies in the fact that it can be standardised and so allows objective coding of clearly defined behavioural reactions. On the downside, battery-style tests are strongly affected by the context in which they are performed [33–34] and it is possible that reducing a suite of behaviours to raw behavioural elements may cause the overall quality of the subject’s behaviour to be lost [34–35]. In contrast, individual rating (via questionnaires) relies on the owners’ or care-takers experience of the dogs’ behaviour, which may be associated with biases or different interpretations by different raters (e.g. [36]). Nonetheless, evidence suggests that questionnaire data can be accurate and consistent [37] and can have some predictive validity (e.g. [38]).

Likewise, personality traits obtained in different studies diverge widely (reviewed in [39]). For instance, most tests for assessing shelter dogs primarily measure social behaviour towards humans and conspecifics and dogs’ tolerance to challenging situations such as being touched on different body parts, and being disturbed when eating etc. (e.g. [27,29,40]). Based on such tests, De Palma et al. [41] identified five traits: Subordination/aggressiveness, Intraspecific dominance-activity, Anxiety-sociability towards dogs, and Playfulness and Sociability towards humans. On the other hand, for working dogs, traits such as “Sharpness (“a dog’s ability to react in an aggressive way towards a serious or serious-looking attack”, [30], p. 120), boldness [32], fearfulness [42], but also willingness to retrieve in puppies [6,24] were suggested to be of relevance. Accordingly, Svartberg and Forkman’s [31] factor analytical study based on a personality test for working dogs found five traits—Playfulness, Chase-proneness, Curiosity/Fearlessness, Sociability and Aggressiveness—and one higher-order, broader dimension, interpreted as a shyness–boldness continuum. Meanwhile, by employing methods used in human psychology, Gosling et al. [43] identified four traits as equivalent to four of the five human personality traits in the five-factor model used in human psychology: Energy (c.f. the human personality factor Extraversion), Affection (c.f. human Agreeableness), Emotional Reactivity (c.f. human Neuroticism) and Intelligence (c.f. human Openness/Intellect).

As mentioned, most existing dog personality tests had very specific (practical) goals in mind and so focused only on a limited aspect of dogs’ personality. So far, to our knowledge, no behaviour test has been specifically developed and validated in order to evaluate pet dog personality in general, rather than to fulfil the more narrow goals of identifying problematic/aggressive behaviours [25–29], suitability as a working dog [5,30], or improving the results of adoption [41]. Moreover, to date, pet dogs (i.e. dogs kept primarily as companions) have largely been neglected in the *experimental* study of dog personality (but see [17,19]. Although several personality questionnaires have been developed for this target group [37,44–47].

Therefore, the aim of the study was to create a comprehensive test of dog personality that goes beyond evaluating problematic (aggressive or fearful) tendencies, or traits that are relevant for working dogs, by exposing pet dogs to a range of situations that they might encounter in their every day life. Based on previous reviews of dog personality [39,48], and our own literature screening, the most common personality factors found in different studies—aside from aggression—were related to four main dimensions: Reactivity (a.k.a. Emotional stability, Neuroticism), Sociability (a.k.a. Agreeableness, Affection), Activity (a.k.a. Extraversion, Excitability), and Trainability-playfulness (a.k.a. Openness, Responsiveness to training). Thus, when designing our method, these four aspects of dog personality were of main interest to us. However, we also aimed at measuring additional aspects of the dogs’ consistent behaviour that were relevant to cognitive performance and the dog-human relationship, such as problem solving ability, frustration tolerance and dependency on their owner, which may relate to a possible fifth dimension Independence-persistence.

Even though the effects of personality on cognition have been demonstrated in numerous studies on nonhuman animals (reviewed by [11]) and despite very early research by Ivan Pavlov (1941 as cited by [11]) demonstrating that personality affects learning in dogs, the potential influence of such individual differences has largely been ignored in cognitive experiments on dogs to date (but see [49]). In addition, previous dog personality assessments have mostly disregarded the role of the human counterpart, although studies have shown that dogs’ behaviour is highly influenced by the behaviour of their owner [50,51], and thus the presence or absence of the owner affects the outcome of temperament testing in dogs [52].

By definition, personality refers to “consistent differences between individuals in their behaviour across time and contexts” [53]. Thus, the assessment of internal consistency (consistency across situations) and test–retest reliability (consistency over time) is what differentiates a personality measurement from a simple behavioural assessment. In a recent (2015) review of studies on personality in pet dogs, some aspects of reliability and validity were measured in 56.82% and 70.45% of studies, respectively [11]. However, measures of internal consistency (the agreement between individual items of measures designed to assess the same theoretical construct) and intra-observer reliability are often not included, while inter-observer reliability, although more often reported, varies widely between studies [39]. Results from test-retest reliability have been very mixed, but generally some consistency over time is observed (reviewed in [18,34,39]).

In creating a personality test, the Vienna Dog Personality Test (hereafter: VIDOPET), tailored specifically to pet dogs, we aimed to produce a more comprehensive evaluation of the validity and reliability across variables, coders, time, testers, and locations. We used factor analytical techniques to identify personality traits in dogs, then assessed the internal consistency and test-retest reliability of the obtained factors, examined intra- and inter-observer reliability, and evaluated effects of test location and test person. Additionally, we aimed to appraise construct validity by correlating results from video coding with video rating, and with owner questionnaires outcomes.

## Materials and methods

### Ethics statement

The conducted research was based on non-invasive procedures for assessing dogs’ behaviour, and such non-invasive observational studies are allowed to be conducted without any special permission in Austria (Tierversuchsgesetz 2012–TVG 2012). The behaviour test was no longer than one hour with a short break in the middle, and the experimental procedure was discussed and approved by the institutional ethics and animal welfare committee at the University of Veterinary Medicine Vienna (Approval numbers: 09/04/97/2012, 04/05/97/2012, 09/10/97/2012, 09/06/2015) in accordance with Good Scientific Practice guidelines and national legislation (http://www.vetmeduni.ac.at/fileadmin/v/z/forschung/GoodScientificPractice_English.pdf). The owners participated in the test on a voluntary basis, they were informed about the purpose and procedure of the test before the onset of the experiment, and they all signed an informed consent form permitting their dogs to participate in the study, and allowing us the use of the recorded data in publications.

### Subjects

Subjects were 217 privately owned Border collies (43.3% males) living in or near Vienna. The dogs’ age ranged from 0.5 years– 15 years (mean age + SD = 4.04+3.50 years).

To obtain a measure of test-retest reliability, the owners and dogs who were still available 2.5–4.7 years (average: 3.8 years) after the first test session were contacted, and N = 37 agreed to participate in a second test session (for more details, see Reliability analyses chapter).

### Room and equipment

**Fig 1:**
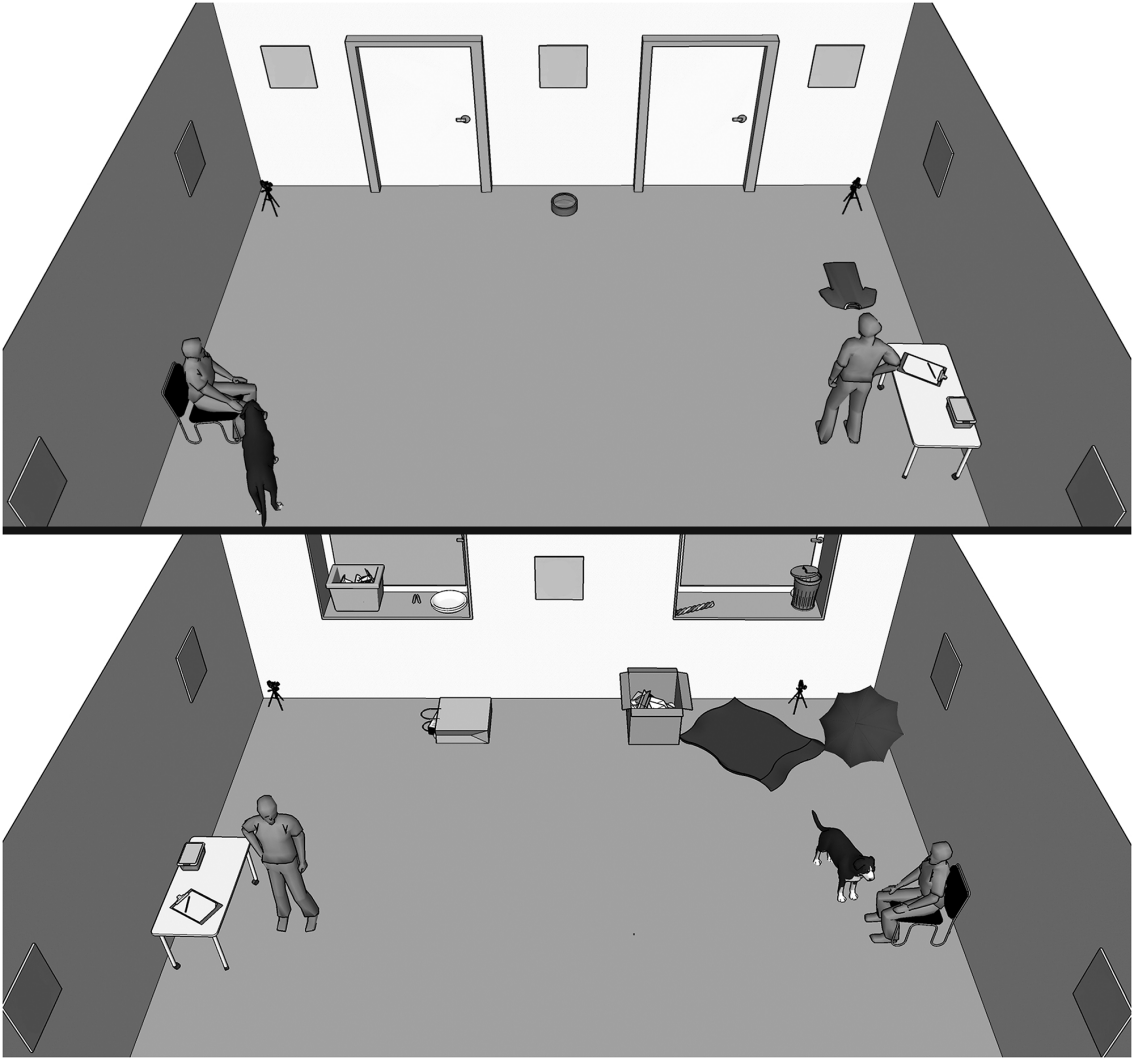
Room setup viewed from the direction of the windows (above), and the doors (below).

The tests took place in one of two experimental rooms; one measured 5 x 6 m, the other 7.2 x 8 m. Equipment in the rooms included a variety of items to explore: a big cardboard box, filled with cardboard scrapings and paper, an old t-shirt on the floor, an opened umbrella in a corner, a water bowl, a bag filled with magazines and a tennis ball, and a large plastic bag, as well as a small table (used for storing some smaller items needed for the test), and a chair (Fig 1). A small plastic bin, a box filled with paper scraps, a tug toy and a tennis ball were stored on two windowsills. Eight pictures of dog faces were displayed on the four walls 1.5 m above the ground. Cameras were positioned in all four corners of the room.

The second test session (used for assessing the test-retest reliability) was carried out in a different test room than the first test session, to ensure that the room was unfamiliar to the dogs.

### Procedure

One of three female experimenters (LW, SR, Claudia Rosam) conducted the behavioural tests, and Judit Berczik performed the retesting of dogs for the test-retest reliability. Most of the subtests were taken or modified from existing dog personality assessments and were selected because behaviours measured in them were found to be related to one or more of the personality dimensions we intended to capture: Reactivity, Sociality, Activity, Trainability-playfulness, Independence-persistence (see details in Table 1). The order of the 15 subtests was the same for all the dogs.

**Table 1:** Hypothesised relationship between the subtests and the personality dimensions we aimed to capture with the test.

	Dimension we aimed to capture				
Subtest	Reactivity	Sociability	Activity	Trainability-playfulness	Independence-persistence
Exploration			x [59,65,66]		
Picture viewing			x [54]		x [41]
Greeting the experimenter		x [31,56,65]			
Food choice					x [58]
Focus & Frustration test	x *				
Separation	x [67]		x [45,59]		x [68]
Greeting after separation		x [56]			
Problem solving I (cage)				x [56]	
T-shirt	x *				x *
Obedience	x [28]			x [45,62]	
Threatening approach	x [69]				
Post-threat interaction		x [56]			
Problem solving II (Bin)				x [64]	
Novel object	x [70–72]		x [65]		x [31]
Ball play				x [28,31]	

An ‘x’ in a cell sign indicates that a given subtest is expected to measure behaviours related to that given dimension. The study on which we based that expectation (if any) is indicated in superscript.

* The protocol of the subtest was our own idea.

1. Exploration (see [54])

Aim: to assess the dogs’ general activity and exploration when placed in an unfamiliar room. The owner (hereafter ‘O’) and the leashed dog entered the room. In the centre of the room, O took off the lead and released the dog, ignoring the dog thereafter. For 60 s, the dog was free to explore the room.

2. Picture viewing (see [54])

Aim: to assess both activity and movement relative to the owners’ movement (following him/her or moving independently).

Upon an auditory signal, O walked to the table and picked up a clipboard with a sheet of paper, which contained a list of eight emotional terms (e.g. fear, anger, etc.). O was instructed to walk around the room, stopping to look at the pictures on the wall, and note down which of the eight dog pictures on the walls corresponded to each emotional term. During the 60 s test, O completely ignored the dog.

3. Greeting the experimenter (see [55–57])

Aim: to assess the dogs’ reaction to an unfamiliar friendly person.

O and the leashed dog stood in the middle of the test room when the experimenter (hereafter ‘E’) entered the room, approached the dog-owner pair, and said “*Hello*” to the owner and the dog. E stopped in front of the dog (just outside the reach of the lead).

- If the dog approached E while wagging its tail or showed neutral behaviour (no avoidance or aggression), E stepped towards the dog, and petted it while continuously speaking to the dog in a friendly way. Then E stepped 1 m sideways within reach of the lead. If the dog followed, E petted the dog again. If the dog did not follow, E crouched down and called the dog again, but did not make contact unless the dog initiated it.
- If the dog showed avoidance behaviour (such as freezing, turning its head away, or moving away), E crouched down and called the dog. If the dog approached E in a non-aggressive manner, E followed the procedure above. If the dog did not respond, E did not try to make direct contact with the dog; instead, she ignored the dog and talked to the owner for 30 s.
- If the dog growled or barked at E, she remained out of reach of the lead and talked continuously to the dog in a friendly manner for 10 s while avoiding eye contact and then terminated the test (note, that none of the tested dogs in the current study responded in this manner).

4. Food choice (modified from [58])

Aim: to assess the dogs’ dependence on the owner by analysing how much the owner’s choice influences the dog’s choice.

The dog’s lead was tied to a hook on the wall, next to the chair of the owner, who was instructed to fill in a questionnaire and ignore the dog. After pre-training, in which dogs had the opportunity to obtain a piece of sausage from a single plate, the food choice test was conducted in two phases.

Phase 1 (Free-choice phase; six trials): E put a piece of sausage on one of the two identical plates (out of sight of the dog), approached to within 1.5 m of the dog and placed both plates on the ground simultaneously (1.5 m apart from each other). Then E moved behind the dog and, after the dog had clearly looked at both plates (ca. 5 s), she released the dog. As soon as the dog had chosen one plate (i.e. approached the plate to within 10 cm), E removed the non-chosen plate. This procedure was repeated six times, and the location of the food was alternated in each trial. The starting position of the baited plate was counterbalanced between dogs.

Phase 2 (Preference phase; six trials): E placed and positioned the plates the same way as during the first phase, but before the dog was released to choose, the O stood up, went to the empty plate, crouched down and pretended high interest in the non-baited plate, repeating “mmm that is yummy!” for approximately 5 s. After O had returned to the chair, E released the dog to make its choice. This procedure was also repeated six times, and the location of the food was alternated in each trial.

Between tests 4 and 5, E took a DNA sample from the dog by rubbing a pair of cotton swabs along the inner side of the dog’s mouth, repeating the procedures on both sides (see [16]). This part of the test sequence was not used in the analyses of the current study.

5. Focus & Frustration test

Aim: to assess the dogs’ persistence and frustration behaviours when trying to obtain an inaccessible food reward.

The dog’s lead was tied to a hook on the wall, next to the chair of the O, who ignored the dog during the test. To increase the dog’s motivation to obtain the sausage during the following test, E started by moving a small piece of sausage in front of the dog at floor level from left to right or from right to left (counterbalanced between subjects) three times, then put the piece of sausage in front of the dog for it to eat. This procedure was then repeated in the other direction. During the test, E stood in front of the dog, turned sideways and did not look directly at the dog. E swung a piece of sausage (approximately 8 cm long), attached to a 30 cm long string, in front of the dog’s nose just out of its reach. After 60 s, E gave a piece of sausage to the dog.

6. Separation (modified from [59])

Aim: to assess the dogs’ reaction to separation, activity when alone, and dependence on the owner.

The dog was released and free to move in the room. Using different doors, O and E simultaneously left the room, without looking at or addressing the dog. The dog was left alone in the room for 60 s.

7. Greeting after separation (modified from [59])

Aim: to assess the dogs’ reaction to a friendly person during the absence of the owner as well as their playfulness.

E entered the room, and then stood next to the door for 5 s without interacting with the dog. If the dog did not approach during this time, she called the dog and greeted it (following the protocol of the Greeting the experimenter test). After the greeting, E initiated a tug-of-war play with the dog for 30 s using the toy on the windowsill. Then E returned the toy to the sill and left the room. After 5 s the owner entered the room and repeated the same procedure as the experimenter (stopping next to the door, greeting the dog, and playing).

Between tests 7 and 8 a break of approximately 5 minutes was taken during which the dog remained outside of the test room.

8. Problem solving I (cage) (see [60,61])

Aim: to assess the dogs’ problem solving ability as well as persistence and frustration when trying to obtain an inaccessible food reward.

E placed a 75 x 55 x 60 cm wire cage in the centre of the room.

Trial 1: After giving the dog the opportunity to explore the cage, E fixed a piece of sausage to the end of a 32 cm long strap and placed the baited end of the strap inside the cage with the other end of the strap sticking out of the cage. E stepped away from the cage, and the O, who sat on a chair ca. 1.5 meters away from the cage, released the dog. During the first 30 s, the O was allowed to encourage the dog verbally, without leaving his/her chair. The test was terminated a) if the dog pulled out the strap and obtained the food, b) if the dog did not interact with the cage for 60 s, or c) after 5 minutes. If the dog was unable to get the food, the experimenter subsequently gave it to the dog.

Trial 2 (blocked trial): If the dog had successfully pulled out the strap in Trial 1, a blocked trial was initiated. E, turning her back to the dog, fixed another piece of sausage to the strap and placed the baited strap in the cage as in Trial 1; however, this time she attached the strap to the cage so that it could not be pulled out. Then the same procedure was followed as in Trial 1 except that the dog was unable to obtain the food.

9. T-shirt test

Aim: to test the dogs’ reaction to an unknown situation of handling/mild restraint.

O put a T-shirt on the dog, without talking to the dog or giving it any instructions. Once the T-shirt had been put on and fixed in position by tying it into a small knot on the dog’s back, O stood up and slowly walked around the room once, looking at the pictures on the wall while ignoring the dog for ca. 30 s. Then O called the dog and took off the T-shirt.

10. Obedience (modified from [62])

Aim: to assess the dogs’ obedience and distractibility.

At the far end of the room, O asked the dog (off lead) to sit, then to lie down and stay. Meanwhile, at the opposite side of the room, E was rustling in a box filled with crumpled newspaper, acting as if searching for something. After giving the stay command, O walked over next to E’s position and stood there facing the dog for 15 s, then called the dog. If necessary, the commands were repeated.

11. Threatening approach (see [63])

Aim: to assess the dogs’ reaction to a threatening person.

O and the leashed dog stood at the far side of the test room, with O holding the lead loosely so that s/he was one step behind the dog. O was instructed not to talk to or interact with the dog. E called the dog’s name, and then started to approach the dog slowly and haltingly, with a slightly bent upper body while staring steadily into the eyes of the dog. The approach was terminated when a) E had reached the dog, b) the dog approached E in a friendly manner, c) the dog reacted aggressively (e.g. growling, barking, or jumping in the direction of the experimenter), or d) the dog retreated behind the owner.

12. Post-threat interaction (see [63])

Aim: to assess the dogs’ behavioural flexibility and reaction to a friendly person after she threatened the dog.

Following the threatening approach, E resolved the situation by stepping back a few steps, crouching sideways and calling the dog in the friendly way. If the dog approached, E followed the protocol of the Greeting the experimenter test. If the dog did not approach, E talked continuously to the dog in a friendly manner for 10 s and then terminated the test.

13. Problem solving II (Bin)

Aim: to test the dogs’ problem solving and learning capability.

The dog’s lead was tied to the hook on the wall. A small plastic bin (31 cm high, 22 cm diameter) was placed 1.5 m in front of the dog. The brim of the lid had been extended by cardboard and tape to a diameter of 31 cm to make lifting it easier for the dogs. O called the dog’s attention and repeatedly demonstrated (ostensive demonstration: [64]) how to remove the lid of the bin to obtain a piece of sausage, which was placed inside the bin. After the fourth demonstration, O put the lid back on the bin and stepped one step back. E removed the dog’s lead and O verbally encouraged the dog to manipulate the bin and obtain the sausage. If the dog was not able to get the food within 60 s, E gave it to the dog.

14. Novel object

Aim: to assess the dogs’ reaction to a startling stimulus as well as their excitability and dependence on the owner.

While O and the dog (off lead, but held at the collar/harness by the owner) turned their backs to E, she placed a 30 x 15 x 10 cm battery-operated toy dog in the middle of the room and turned it on, then stepped back to the wall and stood still. The toy started rolling on the floor and made a noise (resembling human laughter) for 30 s. Upon hearing the noise from the toy, O released the dog, and then stood motionless for 60 s. The toy had an automatic motion sensor that was activated by nearby movement (e.g. the exploring dog), re-initiating the toy’s movement and noise. If the toy did not turn on a second time due to the dog not coming close enough to activate the motion sensor, E turned it on again by walking past it across the room. After 60 s, E turned the toy off, and O showed the turned-off toy to the dog to resolve the situation.

15. Ball play (modified from [62])

Aim: to assess the dogs’ playfulness.

O threw a tennis ball across the room three times. During the first two throws, O encouraged the dog to retrieve the ball and give it back to him/her. After the third throw, O stopped interacting with the dog, stood still and ignored the dog for 15 s. Then O took the ball, placed it on the windowsill and walked around the room while ignoring the dog.

### Behaviour variables

All tests were video-taped, and Solomon Coder beta program (András Péter; www.solomoncoder.com) was used for later analysis of the videos.

#### Video coding

Given that the coding method (assessment of explicit elements of the animals’ behaviour in specific situations) may be more objective in comparison to ratings because it is not influenced by the observers’ subjective interpretations [73], we decided to use a coding approach as our main method of measuring dogs’ behaviour in the test. Coding was performed by one of the four coders (SR, Claudia Rosam, Stephen Jones for the original test, and Judit Berczik for the re-test).

We used a top-down approach by initially coding 160 variables that measured almost every potentially relevant aspect in the different subtests. This list of variables was reduced in several steps as described below, resulting in a set of 70 variables that were used in the subsequent analyses.

Step 1: We removed redundant variables (e.g., when a behaviour was measured both as a frequency and as a duration only one was kept). For infrequently occurring variables that should theoretically be related, composite scales were created (for instance, in stressful or frustrating situations, the variables coding different behavioural expressions of stress/frustration (e.g. vocalisation, yawning, and pacing, etc.) were added up to form a composite scale). Step 1 resulted in a reduction by 53 variables.

Step 2: Variables for which more than 20% of the dogs had missing values were excluded (6 variables).

Step 3: Continuous variables (durations, latencies, and frequencies) with extremely low standard deviation, or extremely high skewness or kurtosis were excluded. For nominal scores, variables where more than 80% of the dogs received the same value were also omitted. This led to the exclusion of another 19 variables.

Step 4: 12 more variables were removed due to very low inter- or intra-observer reliability at the variable level (Cohen’s Kappa or ICC < 0.5). (The results of the variable-level reliability analyses are not shown due to space constraints).

The remaining 70 variables used for the final analysis (see S1 Table) included 30 durations, 12 latencies, 9 frequencies, 1 continuous score (% of correct choices in the food choice test), 3 presence/absence variables, and 15 nominal scores.

#### Video rating

In order to assess how well the selected coded variables captured the behaviour during the test we also developed an alternative ‘video rating’ evaluation method for the videos to compare it to the video coding results. Rating methods (ratings of the overall behavioural disposition of the subjects based on their observed behaviour in specific situations) have been suggested to be more efficient when assessing broader constructs of behaviour due to their integrative nature [74], capturing information not easily viewed when breaking the behaviour down into narrowly defined discrete components during video coding [60]. Thus, we aimed to validate our coding through correspondence with these broader, but potentially more subjective, ratings.

An experienced dog trainer (Sandra Scherner), who was not otherwise involved in the study and was blind to the coding results, rated a subset of 101 videos in a subjective way using an adjective-based rating system. The rating system was created by Sandra Scherner and the last author (SR) on the basis of watching a set of videos (not included in the subset that was later evaluated for comparison with the coding method), and identifying relevant terms to describe the dogs’ behaviour in the respective situations. As a result, 2 to 11 trait-like behaviour characteristics (e.g. “Active”, “Aroused, excited”, “Frustrated”, etc.) were chosen a priori for each subtest (except the Food choice test). The Food choice test was not rated subjectively, since its main outcome was the number of correct vs. incorrect choices. Altogether 76 such subjective variables were rated throughout the test using a 5-point Likert scale from “disagree strongly” to “agree strongly” (S2 Table).

### Dog personality questionnaire

In order to compare the dogs’ behaviour during the test to a more general behavioural assessment, most of the participating dogs’ owners (N = 207) also filled in a personality questionnaire about their dog during the test, the Dog Personality Questionnaire (DPQ-short form, developed by Jones [45]), in German translation (modified by [19]). The questionnaire consists of 45 statements such as “Dog is relaxed when greeting people”, “Dog is curious”, etc. (S3 Table), and measures five personality factors: Fearfulness, Aggression towards people, Activity/excitability, Responsiveness to training, and Aggression towards animals. We chose this questionnaire because of its good psychometric properties, including high internal consistency, inter-observer reliability, test-retest reliability, and predictive validity of the factors [45]. Additionally, a recent publication has demonstrated strong convergence of the DPQ with a different questionnaire, the Monash Canine Personality Questionnaire-Revised (MCPQ-R, [46]), indicating the validity of the underlying canine personality constructs [37]. The questionnaire was translated from English into German and the ratings were made on a 5-point Likert scale (not a 7-point scale as in the original questionnaire, see [19]). In our own previous work, results from the translated questionnaire have shown high temporal consistency [19]. However, to investigate if the factors in the current study were still reliable after these modifications, we analysed the internal consistency (Cronbach’s alpha) of the factors.

### Statistical analyses

#### Data reduction and factor scores

SPSS v. 22 for Windows (IBM Corporation) was used for all statistical analyses. We subjected the 70 coded variables to a two-step data reduction procedure. In the first step, our aim was to reduce the number of variables from each subtest while maximizing the variance retained. In this step, we used a principal component analysis (PCA) with Varimax rotation on the raw variables from each given subtest. We removed no variables from the analyses and retained all principal components with Eigenvalues larger than 1. For the Food choice subtest no PCA was run since the only variable of interest was the change in bias (proportion of correct choices during Phase 2 compared to Phase 1). The 14 subtest-level PCAs on the 70 coded variables resulted in 25 components (S4 Table). In the second step, our aim was to explore the underlying structure of the behaviour across the subtests in order to obtain factors that comprise behaviour in multiple different situations. In this step, we subjected the components extracted in the first step to an exploratory factor analysis (EFA) with Varimax rotation. In this analysis, we decided the number of factors retained by running a Parallel analysis, using the syntax program for SPSS provided by O’Connor [75] and excluded the subtest-components that failed to load with at least 0.3 on any EFA factor in a stepwise manner [76]. These ‘higher-order’ factors were used for further reliability and validity analyses, and we also assessed the independence of the factors using Pearson correlations.

The PCA and EFA results presented a template to calculate the factor scores for each individual dog. In the first step, we standardized the raw variables using z-transformation to control for the different means and minimum/maximum values of the different variable types. In the second step, we calculated the subtest-level component scores by taking the mean of the variables loading with at least 0.4 on a given component (variables that loaded negatively on a component were first multiplied by -1). If a component was comprised of three or more variables, we allowed a maximum of one value to be missing, but if a subject had more than one missing value for a given component, no score was given for that particular component. In the third step, we calculated the ‘higher-order’ factor scores by taking the means of the subtest-level components loading with at least 0.3 on a given ‘higher-order’ factor (components that loaded negatively on a factor were first multiplied by -1). Again, we allowed a maximum of one value out of at least three to be missing.

The data of these second test sessions, and additional codings used for inter- and intra-observer reliability were not included in the original PCA and EFA analyses to avoid pseudoreplication. We calculated the factor scores of these redundant data the same way as the original data, using the PCA and EFA results as templates.

#### Reliability analyses

We analysed six reliability criteria on the EFA factors, for details of the samples and statistics used, see Table 2.

**Table 2:** Sample sizes and statistical tests used for the reliability analyses.

Type of reliability	Sample specifications	Statistical test
Internal consistency	All dogs (*N* = 217)	Cronbach’s alpha
Intra-observer reliability	38 videos were coded twice by the same coder (> 2 years between the two coding sessions).	ICC (2,k absolute agreement)
Inter-observer reliability	40 videos were coded twice by two of three coders.	ICC (1,k absolute agreement)
Test-retest reliability	37 dogs (43.2% males, mean age during first test + SD = 2.76+1.92 years, mean age during second test + SD = 6.53+2.05 years) were tested a second time, on average 3.77 years (range: 2.52–4.72 years) after the first test, by a different experimenter and in a different test room.	ICC (3,k consistency)
Reliability between two test locations	A sample of 72 dogs (36 dogs per room, matched by age and sex) were compared across the two test locations: Room 1: 38.8% males, mean age + SD = 2.53+2.05 years Room 2: 38.8% males, mean age + SD = 2.55+2.02 years	Independent t-test
Reliability between experimenters	A sample of 105 dogs (35 dogs per experimenter, matched by age and sex) were compared across the three experimenters: E 1: 42.9% males, mean age + SD = 1.79+0.90 years E 2: 40.0% males, mean age + SD = 1.66+0.45 years E 3: 45.7% males, mean age + SD = 1.77+0.25 years	ANOVA

E: experimenter, ICC: Intraclass correlation coefficient

#### Validity analyses

We analysed the construct validity of the test by investigating the relationships between the video coding results, the video rating results, and the questionnaire assessments. First, we had to extract broader behavioural constructs (i.e. factors) from the subjective video rating data, for which we used the same two-step data reduction procedure with the same settings as described for the video coding. The 14 subtest-level PCAs on the 76 video rating variables resulted in 29 components (S5 Table) which we then subjected to an EFA. Again, the settings of the EFA were the same as for the coding, except that here we used scree plot visualisation [77] to determine the number of retained factors instead of Parallel analysis. Due to the lower number of subjects in this dataset, Parallel analysis would have resulted in a less reliable factor number. We calculated the factor scores also the same way as described for the video coding, using the PCA and EFA results as templates. To assess the internal consistency of the ‘higher-order’ factors we calculated Cronbach’s alpha values.

Second, when assessing the construct validity, we first made predictions about which video coding, video rating and questionnaire factors are expected to measure overlapping constructs and should therefore correlate with each other, and which factors should be uncorrelated (please refer to the *Predictions* section of the results). Then we analysed the correlations between the video coding factors and the video rating and the questionnaire factors using Pearson correlation, and calculated how many of our predictions were correct (significant) and how many were not. The predicted positive/negative correlations were used to assess convergent validity (i.e., the relationship between theoretically related traits); the cases where we predicted no relationships were used to assess discriminant validity (i.e., the lack of a relationship between theoretically unrelated traits).

## Results

### Video coding EFA results

Based on the result of the parallel analysis we extracted five factors from the EFA, which explained 52.4% of the total variance and were composed of 18 subtest-level components (these latter comprising 52 raw variables from 13 subtests, Table 3). All components from the Threatening approach and the Food choice subtests fell out of the EFA, as they did not load with at least 0.3 on any of the factors.

**Table 3:** Results of the exploratory factor analysis of the video coding.

Subtest component	Raw variables	Sociability-obedience	Activity-independence	Problem orientation	Novelty seeking	Frustration tolerance
Greeting the experimenter	Approach E score	**0.722**	0.135	-0.063	0.062	-0.001
	Greeting E score					
	Tail wagging					
Greeting after separation C1	Approach latency E(-)	**0.665**	0.163	-0.145	0.019	-0.186
	Approach E score					
	Greeting E score					
	Tail wagging E					
	Play Intensity					
Post-threat interaction	Approach E score	**0.610**	0.088	0.051	0.248	-0.054
	Interaction E score					
	Tail wagging					
Obedience C1	Mean latency to obey commands	**-0.456**	0.162	-0.051	0.135	0.027
	Mean recall latency					
	Mean recall latency from cage					
Ball play C1	Follow ball	**0.356**	-0.141	0.137	-0.131	-0.040
	Grab ball					
	Return					
	Give out ball					
T-shirt C2	Move independently	0.007	**0.617**	0.057	0.116	0.032
Picture viewing C1	Move independently	0.034	**0.607**	-0.178	0.147	-0.024
	Explore					
	Look/ follow O(-)					
	Inactive(-)					
Separation C1	Look at door(-)	-0.045	**0.430**	-0.118	0.122	-0.019
	Move					
	Explore					
	Inactive(-)					
Greeting after separation C2	Play Intensity(-)	0.058	**-0.374**	-0.239	0.130	0.061
	Approach latency O(-)					
	Approach O score					
	Greeting O score					
	Tail wagging O					
Exploration	Move	0.062	**0.335**	0.021	0.077	-0.005
	Explore					
	1m from O(-)					
	Look O(-)					
	Inactive(-)					
Problem solving I (cage) C2	Success latency(-)	0.007	0.029	**0.711**	0.124	0.048
	Oriented to Cage					
	Owner 1m(-)					
Problem solving II (Bin)	Oriented to bin	0.024	-0.071	**0.646**	-0.079	-0.175
	Success latency(-)					
Obedience C2	Distract latency(-)	0.084	0.090	0.146	**0.627**	0.018
	Recall latency					
	Mean recall latency from cage					
Ball play C2	Gaze alternate	0.029	0.085	0.036	**0.481**	-0.047
Problem solving I (cage) C1	Oriented to Cage	-0.102	0.148	-0.110	**0.473**	-0.070
	Look O/ E(-)					
	Latency to give up					
Novel object C1	Look O(-)	-0.028	0.065	**-0.320**	**0.412**	-0.042
	Look Toy					
	Look E(-)					
Problem solving I (cage) C3	Vocalisation	0.061	-0.076	0.091	0.044	**-0.643**
	Latency to give up					
Focus & Frustration C2	Snap	0.126	0.118	0.003	0.073	**-0.600**
	Vocalise/ Stress					
Eigenvalue		2.612	2.269	1.745	1.498	1.306
Explained variance (%)		14.510	12.604	9.695	8.323	7.256

Column 1: Subtest components that loaded > 0.3 on at least one ‘higher-order’ factor. If several components were derived from a single subtest, these were labelled sequentially with the name of the subtest followed by C1 (component 1), C2 and C3, respectively. Column 2: Raw variables that made up each subtest-level component. A (-) mark after a variable’s name indicates a negative loading at the subtest-level. E: experimenter, O: owner. These variables were not part of the EFA, they are shown here only to ease the interpretation of the factors. Columns 3–7: Loadings of subtest components on the five ‘higher-order’ factors. Loadings > 0.3 are in boldface. The Eigenvalue and percentage of variance explained for each factor are shown in the last table rows.

We labelled the extracted factors Sociability-obedience, Activity-independence, Problem orientation, Novelty seeking, and Frustration tolerance. Sociability-obedience was composed of 5 subtest-level components (representing 18 raw variables) from 5 different subtests. Items loading strongly on this factor indicate the degree to which a dog is friendly towards strangers, obedient and playful. Activity-independence was composed of 5 subtest-level components (representing 19 raw variables) from 5 different subtests. Items loading on this factor reflect how much the dog moves independently from the owner and explores its environment. Problem orientation was composed of 3 subtest-level components (representing 8 raw variables) from 3 different subtests. Items loading on this factor are associated with the dog’s focus and independent problem solving ability. Novelty seeking was composed of 4 subtest-level components (representing 10 raw variables) from 4 different subtests. Items loading on this factor indicate how interested the dog is in novel objects and distracting stimuli. The final factor Frustration tolerance was composed of 2 subtest-level components (representing 4 raw variables) from 2 subtests, and items loading on this factor indicate how easily the dog gets frustrated in seemingly unsolvable situations.

We found only two weak, but significant correlations between these factors: Novelty seeking correlated positively with Activity-independence, and negatively with Frustration tolerance (Pearson correlation, r = 0.271, N = 208, p < 0.001; r = -0.278, N = 187, p < 0.001, respectively).

### Reliability analyses

Measures of intra-observer and inter-observer reliability of the five factors were good or excellent, and the internal consistency and test-retest reliability were also acceptable (Table 4). The factor scores did not differ significantly between test locations or between experimenters.

**Table 4:** Results of the reliability analyses of the five factors.

	Sociability-obedience	Activity-independence	Problem orientation	Novelty seeking	Frustration tolerance
Internal consistency (Cronbach’s α)					
Cronbach’s α	0.735	0.582	0.607	0.583	0.560
Intra-observer reliability (ICC 2,k)					
ICC	0.969	0.987	0.994	0.951	0.982
F	F_36,36_ = 32.839	F_36,36_ = 80.849	F_37,37_ = 178.124	F_37,37_ = 20.398	F_33,33_ = 54.393
p	< 0.001	< 0.001	< 0.001	< 0.001	< 0.001
Inter-observer reliability (ICC 1,k)					
ICC	0.927	0.962	0.983	0.839	0.915
F	F_39,40_ = 13.729	F_38,39_ = 26.278	F_39,40_ = 60.091	F_39,40_ = 6.224	F_34,35_ = 11.760
p	< 0.001	< 0.001	< 0.001	< 0.001	< 0.001
Test-retest reliability (ICC 3,k)					
ICC	0.614	0.751	0.523	0.481	0.524
F	F_36,36_ = 2.593	F_35,35_ = 4.009	F_36,36_ = 2.095	F_36,36_ = 1.925	F_27,27_ = 2.102
p	0.003	< 0.001	0.015	0.027	0.029
Test location difference (independent t-test)					
t	t_69_ = 0.171	t_69_ = 0.159	t_70_ = 1.497	t_70_ = 0.164	t_66_ = 1.211
p	0.865	0.874	0.139	0.870	0.230
Experimenter difference (ANOVA)					
F	F_2,102_ = 2.422	F_2,100_ = 0.623	F_2,102_ = 1.967	F_2,102_ = 1.632	F_2,90_ = 1.891
p	0.094	0.538	0.145	0.201	0.157

ICC: Intraclass correlation coefficient

### Validity analyses

We used results from video rating provided by a dog trainer and a questionnaire filled in by the owners as secondary measurements to assess the construct validity (i.e. the agreement between different behavioural measurements) of the video coding results.

#### Video rating EFA results

In the video rating EFA analysis, we identified five factors based on the Scree plot, which we labelled Sociability-activity, Calmness, Excitability, Placid disposition, and Distractibility. The five factors explained 53.2% of the total variance and were composed of 23 subtest-level components (these latter comprising 68 raw variables from 13 subtests, Table 5). No rating was made for the Food choice test, and all components from the Problem solving I (cage) subtest fell out of the EFA, as they did not load with at least 0.3 on any of the factors. The Cronbach’s alpha values indicated acceptable internal consistency for all factors (Table 5).

**Table 5:** Results of the exploratory factor analysis of the video rating.

Subtest component	Raw variable	Sociability-activity	Calmness	Excitability	Placid disposition	Distractibility
Post-threat interaction C1	Friendly	**0.743**	-0.161	0.058	0.290	-0.014
	Aroused, excited					
	Interested in E					
	Passive(-)					
Greeting the experimenter C1	Aroused, excited	**0.741**	-0.175	0.076	0.241	-0.147
	Interested in E					
	Passive(-)					
	Greeting intensity					
Greeting after separation C2	Relaxed E	**0.671**	**0.410**	-0.080	-0.220	0.003
	Aroused, excited E					
	Interested in E					
	Passive E(-)					
	Greeting intensity E					
T-shirt C2	Passive	**-0.538**	-0.030	-0.129	0.135	0.017
	Stressed(-)					
Exploration	Dependent(-)	**0.417**	0.025	**0.372**	-0.030	0.185
	Active					
	Interested in surroundings					
	Aroused, excited					
Ball play C1	Ball motivated	**0.320**	0.072	0.298	-0.077	-0.155
	Aroused, excited					
	Playfulness					
Post-threat interaction C2	Friendly	-0.015	**0.645**	-0.025	0.018	-0.212
	Relaxed					
	Interested in E					
Greeting the experimenter C2	Relaxed	-0.056	**0.632**	0.096	0.105	-0.004
	Interested in E					
Greeting after separation C3	Relaxed O	**-0.432**	**0.577**	-0.227	-0.101	**0.563**
	Aroused, excited O(-)					
	Relaxed E					
	Aroused, excited E(-)					
Picture viewing C2	Confident	0.052	**0.477**	-0.214	0.134	0.020
	Relaxed					
	Aroused, excited(-)					
Separation C2	Active	0.116	**0.374**	0.283	-0.078	-0.033
	Focused on door(s)(-)					
Picture viewing C1	Dependent(-)	**0.340**	-0.014	**0.573**	0.052	0.077
	Active					
	Interested in surroundings					
	Aroused, excited					
Separation C1	Relaxed(-)	0.189	**-0.353**	**0.568**	-0.074	0.235
	Aroused, excited					
	Stressed, frustrated					
Focus & Frustration C1	Active	0.183	-0.109	**0.535**	-0.005	-0.226
	Focused					
	Motivated					
	Relaxed(-)					
	Aroused, excited					
Ball play C2	Cooperation	0.175	-0.019	**-0.432**	-0.014	-0.028
	Inviting to play					
Threatening approach C2	Appease	0.082	0.125	**0.410**	0.279	0.005
	Watchful					
	Offensive approach					
	Avoidance behaviour					
Greeting after separation C1	Relaxed O	0.243	-0.216	0.078	**0.630**	0.292
	Aroused, excited O					
	Interested in O					
	Passive O(-)					
	Greeting intensity O					
	Appease O(-)					
Focus & Frustration C2	Focused	-0.154	0.100	-0.170	**0.455**	-0.121
	Relaxed					
	Frustrated(-)					
Problem solving II (Bin)	Problem solving ability(-)	0.073	0.127	0.156	**0.449**	0.225
	Asks for help					
T-shirt C1	Relaxed	-0.131	**0.317**	-0.053	**0.421**	-0.045
	Insecure(-)					
Novel object C1	Active	0.279	-0.070	0.264	**0.340**	-0.077
	Interested in object					
	Dependent(-)					
	Careful(-)					
Novel object C2	Confident(-)	0.019	-0.200	-0.103	-0.014	**0.564**
	Insecure					
Obedience C2	Lay down(-)	-0.073	-0.011	0.109	0.099	**0.502**
	Come(-)					
	Distractible					
Eigenvalue		4.084	2.565	2.097	1.848	1.623
Explained variance (%)		17.756	11.152	9.118	8.036	7.057
Cronbach’s alpha		0.773	0.736	0.645	0.551	0.566

Column 1: Subtest components that loaded > 0.3 on at least one ‘higher-order’ factor. If several components were derived from a single subtest, these were labelled sequentially with the name of the subtest followed by C1 (component 1), C2 and C3, respectively. Column 2: Raw variables that made up each subtest-level component. A (-) mark after a variable’s name indicates a negative loading at the subtest-level. E: experimenter, O: owner. These variables were not part of the EFA analysis, they are shown here only to ease the interpretation of the factors. Columns 3–7: Loadings of subtest components on the five ‘higher-order’ factors. Loadings > 0.3 are in boldface. The Eigenvalue, percentage of variance explained and Cronbach’s alpha values (assessing the internal consistency) for each factor are shown in the last table rows.

#### Questionnaire

The internal consistency (Cronbach’s alpha) of the five questionnaire factors in the current sample ranged from 0.646 (Responsiveness to Training) to 0.793 (Fearfulness) (S3 Table). While these values were slightly lower than the internal consistency measures of the original English questionnaire (ranging from 0.742 to 0.838, [45]), they were still acceptable, confirming that the translation of the questionnaire from English to German, and the modification of the rating scale (from a 7 point to a 5 point Likert scale), did not cause marked changes in the factors’ structure.

#### Predictions

As the video rating system was developed independently from the coding system, the video coding factors only partially overlapped in their content with the video rating factors. The behaviour test could also not cover all aspects of the questionnaire factors (which included questions such as fear at the vets or chasing small animals that were not investigated during the behaviour test). Therefore, we made only broad predictions regarding the expected theoretical relationships and lack of relationships between the factors (Table 6).

**Table 6:** Predicted relationships between the five video coding factors and the video rating factors and questionnaire factors.

	Video coding factors				
Video rating factors	Sociability-obedience	Activity-independence	Problem orientation	Novelty seeking	Frustration tolerance
Sociability-activity	**+**	**+**			
Calmness	**+**	**+**			**+**
Excitability		**+**		**+**	**-**
Placid disposition		**+**	**-**	**+**	**+**
Distractibility					
**Questionnaire factors**					
Fearfulness	**-**				
Aggression towards people	**-**				
Activity, excitability	**+**	**+**		**+**	
Responsiveness to training				**+**	
Aggression towards animals					

A ‘+’ sign indicates an expected positive correlation, and ‘-’ a negative correlation. In the cases of the cells left empty, no significant relationship was predicted

#### Video coding vs. video rating

Regarding convergent validity, our results indicated that 10 of the 12 predicted correlations (83.3%) between the video coding and the video rating factors were significant, and in the predicted direction (as indicated in Table 6). Two of the predicted correlations (Placid disposition rating factor vs. Activity-independence and Frustration tolerance coding factors) did not reach statistical significance (Table 7).

**Table 7:** Pearson correlations between the video coding, video rating and questionnaire factors.

	Video coding factors				
Video rating factors	Sociability-obedience	Activity-independence	Problem orientation	Novelty seeking	Frustration tolerance
Sociability-activity	0.641***	0.497***	-0.140	0.328**	-0.302**
Calmness	0.226*	0.327**	-0.044	-0.028	0.285**
Excitability	0.074	0.420***	-0.242*	0.353***	-0.482***
Placid disposition	0.073	**-0.021**	-0.594***	0.385***	**0.006**
Distractibility	-0.181	0.053	-0.128	-0.151	0.104
**Questionnaire factors**					
Fearfulness	-0.175*	-0.029	-0.140*	-0.040	0.130
Aggression towards people	-0.218**	-0.061	-0.051	0.013	0.037
Activity, excitability	0.215**	0.194**	-0.059	0.226**	-0.226**
Responsiveness to training	0.050	-0.103	0.069	*-0*.264***	0.076
Aggression towards animals	-0.129	-0.041	0.191**	-0.119	0.113

Predicted correlations are presented in bold. The correlation between Novelty seeking and Responsiveness to Training factors is also marked in italics because the correlation we found was in the opposite direction than we predicted.

* p < 0.05;

** p < 0.01;

*** p < 0.001

Regarding discriminant validity, in 13 cases, we expected no significant relationship between the coding and rating factors, and in 10 cases (76.9%) our predictions were correct. Three unpredicted correlations were found: The Sociability-activity factor obtained by the rating method correlated positively with Novelty seeking and negatively with Frustration tolerance coding factors, while Excitability (rating factor) correlated negatively with Problem orientation (coding factor) (Table 7).

#### Video coding vs. questionnaire

Five of the six predicted correlations between the video coding and questionnaire factors were significant and in the predicted direction (83.3%). However, instead of the predicted positive relationship between the Novelty seeking coding factor and the Responsiveness to Training questionnaire factor, we found a significant negative correlation between these factors (Table 7). From the 19 cases where we predicted no significant relationships, 16 were indeed nonsignificant (84.2%). We found three unpredicted correlations: the Problem orientation coding factor correlated negatively with Fearfulness and positively with Aggression towards animals questionnaire factors while Frustration tolerance (coding factor) correlated negatively with Activity-excitability (questionnaire factor) (Table 7).

## Discussion

The current study aimed at developing a reliable and valid personality test for pet dogs. The VIDOPET yielded five personality factors, each comprising the dogs’ behaviour in multiple situations, and test results were not significantly affected by testing location, test person or coder identity. Test-retest analysis indicated high consistency of the factors over a period of 2.5–4.5 years. Significant correlations between the test results and the video ratings and owners’ assessments demonstrated the construct validity of the VIDOPET. Based on these results, this test battery proved to be a suitable method to investigate dog personality.

While most of the existing dog personality tests are designed for working dogs and shelter dogs, where there is a particular interest in identifying fear and aggression issues, we were interested in the general personality profile of pet dogs, and accordingly, the focus of the VIDOPET was directed more at assessing five dimensions of personality: Reactivity, Sociability, Activity, Trainability-playfulness, and Independence-persistence. However, according to the results of our statistical tests, the five factors we found based on the video coding only partly overlapped with the traits we intended to capture. Sociability and the Obedience/Playfulness part of the Trainability-playfulness dimension ended up as one single factor we labelled Sociability-obedience. Activity and the Independence part of the Independence-persistence dimension ended up as one single factor we labelled Activity-independence. The Reactivity dimension seems to have divided into two aspects: firstly, a factor comprising interest in novel stimuli and in appetitive stimuli (food, ball)–labelled Novelty seeking—and secondly, emotional reactions when the desired goal cannot be reached—labelled Frustration tolerance. The fifth factor we found was labelled Problem orientation, which appears to be related to independent problem solving, and so may represent another part of the Independence-persistence dimension.

There is also some overlap between our factors and other theoretical personality structures (especially those with less focus on practical applications). For example, the first three factors of VIDOPET are comparable to the four traits identified by a modified version of the human Big Five Inventory (BFI; see [78]) by Gosling et al. [43]. Energy (cf. human Extraversion) can be compared to our Activity-independence, Affection (cf. human Agreeableness) to Sociability-obedience, and Intelligence (cf. human Openness/ Intellect) to Novelty seeking and possibly Problem orientation. Only Emotional Reactivity (cf. human Neuroticism) did not emerge clearly in the current test. This could be due to the fact that the test was not designed to put the subjects under severe stress, where individual variation in such a trait would become most apparent. In comparison to previous dog personality assessments, which typically exposed dogs to a range of potentially fear- or stress-inducing situations, most of the situations we hypothesized to measure reactivity (including short frustration, mild restraint, novel objects and distracting stimuli), were relatively mild. Only the threatening approach and the separation from the owner could be expected to cause more pronounced stress responses in some individuals. However, these two situations would likely evoke very different emotional reactions and so no consistent emotional reactivity factor was found.

There were only two weak correlations between these factors, supporting the existence of separate personality traits.

### Reliability

Given the relatively small number of items per factor, the internal consistency of the factors was found to be acceptable (with Cronbach’s alpha values ranging from 0.55 to 0.77 [79,80]), as lower Cronbach’s alpha values often ensue with a lower number of items [81]. Moreover, unlike most of the studies in this field, our factors were created from different types of variables (durations, latencies, frequencies and nominal scores), all with different distributions, which could also have contributed to relatively lower Cronbach’s alpha values.

Both intra-observer and inter-observer reliability of the factors were excellent. Compared to previous studies (reviewed by [5]), inter-observer reliability was extremely high. This probably reflects the objective nature of the coding system, with little error generated by differences in subjective appraisal by the coders, as well as the fact that we excluded raw variables with low inter- or intra- observer reliability.

The VIDOPET had high test-retest reliability, especially considering the long interval between the tests (mean: 3.8 years), and that the test location, experimenter, and coder were not balanced between test and retest. However, we found no significant effects of location or experimenter on any of the factors in the sample selected for comparison, indicating that the test is reliable across environments and with different (female) experimenters. Our test-retest reliability is only a little lower than that measured in a different dog personality test, the Dog Mentality Assessment, over a time period of only 30–35 days [82]. To our knowledge, only one study found an even higher consistency over a longer time frame (greater than 6 years) for the trait impulsivity in dogs [83]. On average, personality consistency in adult dogs has been reported to be lower than in the current study, according to a meta-analysis by Fratkin et al. [18], even more so for between-test intervals classified as ‘long’ (> 24 weeks; see also [5,82]). One explanation for the comparatively high reliability in our study could be the excellent inter-observer agreement, as lower consistency estimates will ensue when inter-observer reliability is poor. The factor with the highest test-retest reliability was Activity-independence. In a previous study, exploratory activity (a trait akin to Activity-independence) was the only trait to be significantly correlated when dogs were tested as puppies (6–7 weeks) and then retested as adults (1.5–2 years) [83]. This supports the notion that activity/exploration in a novel environment is one of the most consistent traits in dogs. The lowest test-retest reliability was found in the Novelty seeking factor. Since the same ‘novel’ objects and stimuli were used in test and retest for the subtests included in this factor, it could be expected that they provoked slightly different reactions the second time. Similarly, Svartberg et al. [82] also suggested that their Curiosity/Fearlessness trait (akin to our Novelty seeking trait) is sensitive to the novelty of the object/situation. This suggests that the test is less repeatable for this factor, but not necessarily that the factor itself has lower consistency. Retesting with different stimuli in the re-test would be a way to differentiate between these possibilities.

### Validity

The construct validity of the test was investigated by correlating the personality factors extracted from analysis of the video coding to two other personality assessments: video ratings by a dog trainer and questionnaire ratings of the owners. In both comparisons, more than 80% of the predicted correlations were significant (and in the predicted direction), and more than 75% of the cases where no significant relationship was predicted, were indeed nonsignificant. Overall both convergent validity (factors of different measurements that theoretically should be related to each other correlate together) and discriminant validity (factors of different measurements that theoretically are not related to each other do not correlate together, [34]) according to the methods used, were satisfactory. However, there were also deviations from our predictions. For example, for the coding-rating comparison, we found an unpredicted, although biologically meaningful positive relationship between Novelty seeking and Sociability-activity, and a negative relationship between Excitability and Problem orientation. For the coding-questionnaire comparison, one correlation turned out to be in the opposite direction than we predicted. Instead of a positive relationship, Novelty seeking correlated negatively with Responsiveness to training. The prediction was made based on the assumption that novelty seekers may be more sensitive to reward, and thus in general, more amenable to positive reinforcement training. It is, however, a possibility that novelty seekers, being curious about various environmental stimuli, show a higher tendency to self-reward, or get distracted more easily during training, which would lower the owners’ perception of the dogs’ obedience and trainability.

However, even though the correlation strength between the VIDOPET coding and the rating assessments are at a similar level as those reported in other dog personality studies (e.g. [84,85]) and human studies (e.g. [39,43,86]), the proportion of variance explained is still relatively small. This problem seems to be a general issue when correlating results from different personality instruments, and can be explained by the combinations of three issues (also see [36]):

First, the strength of the correlation between different instruments strongly depends on how much the compared factors overlap in their content. Studies that devised the two instruments to match each other reported higher correspondence between them (see for example [43,45], who both used video rating and questionnaires, or [59], who used video coding and questionnaires). Since this overlap varies from study to study, it could explain the huge variability in the reported correlations between different instruments. Unfortunately, within the confines of our study, it was not a factor we could explicitly measure or control. Second, the behaviour the animals show in controlled environments and in selected standardized situations may not necessarily correspond to the behaviour they show in everyday life (what the owners can observe). Therefore, when contrasting an assessment based on a limited number of test situations, and a questionnaire expressing a general impression of behaviour based on numerous and various situations, their relationships are bound to be relatively weak. These two factors might explain why we found more and stronger correlations between the video coding and video rating factors than between the video coding and questionnaire factors: in the former case, both assessments were based on observations of the same situations. However, even between the two video-based methods the correlations were also only moderate, suggesting that the dissimilarity in the situations observed and in the factor content are not the only explanations for the weaker correlations. The third possible issue is that the coding and the ratings may measure somewhat different behavioural constructs. The raters interpret the dog behaviour based on the whole behavioural sequence observed, and the rating methods use broader, more general descriptors of the behaviour allowing the raters to take into account many different ways a given behavioural characteristic could be expressed [18,73,74]. Contrary to that, coders are restricted to the clearly defined behavioural elements specified in the variable list, dissecting the behaviour into its individual elements [18,40,74]. In harmony with this, studies correlating video rating with questionnaire rating generally reported higher correlations (e.g. [60,87]) than those correlating video rating with video coding (e.g. [84,85]). To summarise, the significant correlations between the different assessments demonstrate the construct validity of the VIDOPET, but the relatively weak relationships warrant caution when interpreting these associations.

### Limitations

Of course, no available tool can represent dog personality as a whole. One limitation of the current test is that it includes no test for assessing specific aspects of the dogs’ behaviour, such as intraspecific interactions or reactions to prey animals. Moreover, only one subtest addresses fearfulness and propensity to display aggression, and for this methodological reason, fearfulness and aggression did not turn out as personality traits in the statistical analysis (since no cross-situational consistency could be demonstrated). Thus, if these particular traits are of interest, adding further subtests may be necessary. Second, bear in mind that to date the test has been validated only in a single breed, the Border collie. Given that piloting of the test was performed on various dog breeds and that the test has been used with a variety of breeds in at least one other study [88], we are positive that the methodology is suitable for investigating personality also in other dog breeds, although, conceivably, factor structure may differ somewhat between breeds.

### Conclusions

We developed a personality test battery for pet dogs, which yielded five personality factors. In part, these factors appear to overlap with personality dimensions akin to those in humans, as reported in Gosling et al. [43] based on a canine adaption of the human Big Five Inventory. However, some novel traits emerged, probably reflecting the somewhat different focus of our study compared to most previous dog personality assessments. To our knowledge, this is the first study to demonstrate that Frustration tolerance and Problem orientation are consistent and can be considered personality traits in dogs. The evaluation of six reliability criteria (including intra-observer reliability, reliability between experimenters, and between test locations), which have rarely been addressed in former studies, demonstrated good reliability of the test, and our results also showed very high consistency of personality traits in adult dogs over a period of several years. Additionally, we demonstrated both convergent and discriminant validity of the test. Therefore, we conclude that the VIDOPET is a highly reliable and valid assessment for measuring personality in pet dogs.

## Supporting information

S1 Table Variables from video coding that remained in the analysis. E: experimenter, O: owner. Durations were calculated as percent of time.
(PDF)

S2 Table Variables used in the video rating. The variables were rated on a Likert scale from 1–5 (“disagree strongly”, “tend to disagree”, “partly-partly”, “tend to agree”, “agree strongly”). E: experimenter, O: owner.
(PDF)

S3 Table The questionnaire used in the study (DPQ short form, Jones, 2008). The questions were rated on a Likert scale from 1–5 (“disagree strongly”, “tend to disagree”, “partly-partly”, “tend to agree”, “agree strongly”), items marked with an asterisk were reverse coded. The internal consistency of factors calculated from the data of the present study are also shown.
(PDF)

S4 Table Results of the subtest-level PCA analyses of the video coding. For definition of the variables see S1 Table. E: experimenter, O: owner. Loadings > 0.4 are in bold.
(PDF)

S5 Table Results of the subtest-level PCA analyses of the video rating. E: experimenter, O: owner. Loadings > 0.4 are in bold.
(PDF)
