# Correction: Personality traits in companion dogs—Results from the VIDOPET

**DOI:** 10.1371/journal.pone.0224199

**Published:** 2019-10-16

**Authors:** Borbála Turcsán, Lisa Wallis, Zsófia Virányi, Friederike Range, Corsin A. Müller, Ludwig Huber, Stefanie Riemer

The Data Availability statement for this paper is incorrect. The correct statement is: The data are uploaded to Figshare database: https://doi.org/10.6084/m9.figshare.5477932.v2.
