# Supplementary material for: Personality traits in companion dogs—Results from the VIDOPET
Source: PLoS One. 2018 Apr 10;13(4):e0195448. doi: 10.1371/journal.pone.0195448 (PMC5892901; doi:10.1371/journal.pone.0195448)
Supplement: S1 Table — E: experimenter, O: owner. Durations were calculated as percent of time. (PDF) [file pone.0195448.s001.pdf]

S1 Table. Variables from video coding that remained in the analysis. E: experimenter, O: owner. Durations were calculated as percent of time.

Subtest	Variable	Definition	Type
Exploration	Move	Locomotion; any movement of the legs leading to a forward or backward motion.	duration
	Explore	Dog’s nose is within 10 cm of the floor or any other surface (e.g., wall, table, objects) or dog has placed both front paws on an elevated surface (e.g., window sill, table). Does not include drinking or lying down.	duration
	1m from O	Dog’s head is within 1m from O.	duration
	Look O	The dog's head is oriented towards the owner (both when the dog is stationary and when moving).	duration
	Inactive	Dog is standing, sitting, or lying in the room, without exploring according to the above definition; also included: transition from standing to sitting or from sitting to lying and scratching.	duration
Picture viewing	Move independently	Moving independently from O (i.e. not moving in the direction of the owner or moving in the same direction as the owner in a proximity of 1m to the owner. Definition of ‘move’ as above.	duration
	Explore	Dog’s nose is within 10 cm of the floor or any other surface (e.g., wall, table, objects) or dog has placed both front paws on an elevated surface (e.g., window sill, table). Does not include drinking or lying down.	duration
	Owner 1m	Dog’s head is within 1m from O.	duration
	Look/follow O	The dog's head is oriented towards O (both while the dog was stationary and when moving), or dog is following the moving O (moving while looking at O or walking behind/ next to O in the same direction as O).	duration
	Inactive	Dog is standing, sitting, or lying in the room, without exploring according to the above definition; also included: transition from standing to sitting or from sitting to lying and scratching.	duration
Greeting the experimenter	Approach E score	When E approaches the dog and O, the dog 0: does not approach the experimenter; 1: approaches hesitatingly or after a while when called; 2: approaches immediately when called; 3: approaches immediately without calling.	nominal
	Greeting E score	When E approaches the dog and O, the dog 0: does not approach E/ approaches initially but then shows avoidance behaviour; 1: approaches E but then behaves passively and does not elicit interaction, with or without tail wagging; 2: friendly greeting, tail wagging, may cuddle up, jump or lick; 3: very excited/ enthusiastic greeting with intensive searching for contact and tail wagging.	nominal
	Tail wagging	When E approaches the dog and O, the dog 0 shows no or very little tail wagging; 1: wags its tail intermittently; 2: wags its tail most of the time.	nominal
Food choice	Change in bias	% correct choices in Food Choice test 2 subtracted from % correct choices in Food Choice test 1.	composite/ continuous
Focus & Frustration test	Oriented to Food	Dog’s head is facing in the direction of the object or E’s hand (swinging the sausage).	duration
	Look E	Dog’s head is facing in the direction of the experimenter's face.	duration
	Snap	Snapping at the food, grabbing with the mouth into the air (may or may not be accompanied by lunging).	frequency

Subtest	Variable	Definition	Type
	Vocalise/ Stress	Frequency of frustration/stress signals including yawning, lip licking, stretching, scratching/ grooming itself, shaking, or vocalisation.	frequency
Separation	Near door	At least one foot of the dog is within 1 m of one of the exit doors (marked by a circle on the floor).	composite/ duration
	Look at door	Dog's head is oriented towards one of the doors while dog is stationary (sitting, lying, standing). Not coded when dog is "near door" (see above).	composite/ duration
	Move	Locomotion; any movement of the legs leading to a forward or backward motion.	duration
	Explore	Dog's nose is within 10 cm of the floor or any other surface (e.g., wall, table, objects) or dog has placed both front paws on an elevated surface (e.g., window sill, table). Does not include drinking or lying down.	duration
	Inactive	Dog is standing, sitting, or lying in the room, without exploring according to the above definition; also included: transition from standing to sitting or from sitting to lying and scratching.	duration
	Vocalise/ Stress	Frequency of stress signals including yawning, lip licking, stretching, scratching/ groom itself, shaking, jumping at the door, scratching the door, and vocalization.	composite/ frequency
Greeting after separation	Approach latency E	After E's entry, latency of approaching E to within 20 cm.	latency
	Approach E score	While E was standing next to door, the dog 0: did not approach E (to within 20 cm); 1: approached hesitatingly or after a while when called; 2: approached when called immediately; 3: approached immediately without calling.	nominal
	Greeting E score	After E's entry, the dog 0: does not approach E/ approaches initially but then shows avoidance behaviour; 1: approaches E but then behaves passively and does not elicit interaction, with or without tail wagging; 2: friendly greeting, tail wagging, may cuddle up, jump or lick; 3: very excited/ enthusiastic greeting with intensive searching for contact and tail wagging.	nominal
	Tail wagging E	While E is standing next to the door, the dog 0: shows no or very little tail wagging; 1: wags its tail intermittently; 2: wags its tail most of the time.	nominal
	Play Intensity	When the experimenter invited the dog to play, the dog 0: did not play; 1: mouthed the toy sometimes, but no real play or needed some encouragement; 2: mouthed the toy and sometimes pulled it/brought it back; 3: was very enthusiastic, played more than 90% of the time.	nominal
	Approach latency O	Latency of approaching O after the separation test.	latency
	Approach O score	0: does not approach O/ approaches initially but then avoids O so no real interaction; 1: approaches O but shows little interest towards O, may behave passively after approaching, with or without tail wagging; 2: friendly greeting, tail wagging, may cuddle up, jump or lick; 3: very excited/ enthusiastic greeting with intensive searching for contact and tail wagging.	nominal

Subtest	Variable	Definition	Type
	Greeting O score	After O's entry, the dog 0: does not approach O/ approaches initially but then shows avoidance behaviour; 1: approaches O but then behaves passively and does not elicit interaction, with or without tail wagging; 2: friendly greeting, tail wagging, may cuddle up, jump or lick; 3: very excited/ enthusiastic greeting with intensive searching for contact and tail wagging.	nominal
	Tail wagging O	While O is standing next to the door, the dog 0: shows no or very little tail wagging; 1: wags its tail intermittently; 2: wags its tail most of the time.	nominal
Problem solving I (cage)	Success latency	In Trial 1, the latency until the strap has been pulled out of the cage completely or the dog has otherwise obtained the sausage.	latency
	Oriented to Cage	Dog's nose or paw is within 10cm of cage and dog is shows an interest in the cage. e.g. sniffing trying to get the food, walking around it while oriented towards the cage. Does not include passively looking at the cage, even when physical contact with the cage is made.	duration
	Look O/ E	Looking at O or E, i.e. the dog's head is oriented towards O or E.	composite/ duration
	Owner 1m	Dog's head is within 1m from owner.	duration
	Vocalisation	Noise produced by vocal chords; barking, whining but not panting or breathing.	frequency
	Latency to give up	Dog does not approach cage for 1min. (marker at the beginning of this period). Coded for the blocked trial if dog has obtained the reward in the first trial, coded for the first trial if dog has been unsuccessful in this trial.	latency
T-shirt	Inactive	After the t-shirt has been put on. Dog is standing, sitting, or lying in the room, without exploring according to the above definition; also included: transition from standing to sitting or from sitting to lying and scratching.	duration
	Look O	Looking at O, i.e. dog's head is oriented towards O while stationary; mutually exclusive with Follow O.	duration
	Follow O	Dog is following the moving O (moving while looking at O or walking behind/ next to O in the same direction as O).	duration
	Move independently	Dog is moving independently from the owner.	duration
Obedience	Mean latency to obey commands	The mean latency to obey the "sit" and "lay down" commands. The time from the first verbal or non-verbal command until the dog sat (rumps on the floor), or lay down completely (whole body on the floor).	composite/ latency
	Distract latency	Latency from the sit command until the dog starts to move towards the distraction (even if the owner manages to call it back).	latency
	Recall latency	Latency to obey the O's recall during the distraction test (the time from the first verbal command until the dog is within 10 cm of the owner).	latency
	Mean recall latency	At six time points during the test, the owner is asked to recall their off-lead dog. Mean latency to obey the "come" command, from the first verbal or non-verbal command until the dog makes the first movement towards the owner (and then actually comes to the owner). Missing data for many dogs – used only when latency data are available from at least two recalls.	composite/ latency

Subtest	Variable	Definition	Type
	Mean recall latency from cage	Mean latency to obey to the "come" command during the Cage test (1. after the dog ate the single piece of sausage in front of the cage; 2. after the dog ate the sausage from the strap). From the first verbal or non-verbal command until the dog makes the first movement towards the owner (and then actually comes to the owner).	composite/ latency
Threatening approach	Latency to aversive reaction	Latency to the first 'aversive' (aggressive or avoidant) reaction to the threatening approach (except looking away) – i.e. moving away, hiding, barking, growling.	latency
	Latency to friendly reaction	Latency to first 'friendly' reaction to threatening approach – e.g. when the dog started tail wagging, and/ or moved towards the experimenter showing behaviours of appeasement.	latency
	Threat reaction	Behaviour shown just before the test is terminated because either the experimenter has reached the dog or the dog has shown a strong reaction. 0: friendly, appeasing, i.e. approach E with tail wagging; 1: passive, i.e. no approach and no avoidance, may look away, and may wag tail intermittently; 2: avoidant, i.e. hid behind the owner/ moved away from the experimenter (with low tail and ear position); 3: aggressive, i.e. bark, growl, snap, and/ or lunge towards E.	nominal
	Bark	During the threatening approach, the dog barks one or more times: 0: no; 1: yes.	1/0
	Growl	During the threatening approach, the dog growls one or more times: 0: no; 1: yes.	1/0
	Approach friendly	During the threatening approach, the dog approaches E in a friendly, appeasing or submissive way (i.e. may wag the tail, with ears back, and/or invite play): 0: no; 1: yes.	1/0
	Move off	During the threatening approach, the dog 0: does not move away from the approaching E; 1: moves away from the experimenter, but does not move behind O; 2: moves behind O.	nominal
Post-threat interaction	Approach E score	Dog's reaction when the experimenter turns sideways, crouches and calls the dog in a friendly voice after the threatening approach: 0: does not approach E within 5 seconds; 0: does not approach E; 1: approaches hesitatingly or after a while when called; 2: approaches when called immediately; 3: approaches immediately without calling or has already approached in a friendly way during the threatening episode.	nominal
	Interaction E score	When E has crouched down post-threat, the dog 0: does not approach E/ approaches initially but then shows avoidance behaviour; 1: approaches E but then behaves passively and does not elicit interaction, with or without tail wagging; 2: friendly greeting, tail wagging, may cuddle up, jump or lick; 3: very excited/ enthusiastic greeting with intensive searching for contact and tail wagging.	nominal
	Tail wagging	When E has crouched down post-threat, the dog 0: shows no or very little tail wagging; 1: wags its tail intermittently; 2: wags its tail most of the time.	nominal
Problem solving II (Bin)	Oriented to bin	Dog's nose or paw is within 10cm of bin, dog is oriented towards bin.	duration
	Success latency	Latency until the cover of the bin has been completely removed.	latency
Novel object	Owner 1m	Dog's head is within 1m from O.	duration
	Toy 1m	Dog's head is within 1m from Toy. If dog is both near the toy and O or E, this is coded as Toy 1m.	duration

Subtest	Variable	Definition	Type
	Look O	The dog's head is oriented towards O.	duration
	Look Toy	The dog's head is oriented towards the toy.	duration
	Look E	The dog's head is oriented towards E.	duration
Ball play	Follow ball	Number of times the dog follows the thrown-away ball.	frequency
	Grab ball	Number of times the dog grabs the thrown-away ball.	frequency
	Return	Number of times the dog returns with the ball to within 1.5m of the owner within 5 seconds after first touching ball.	frequency
	Give out ball	Number of times the dog gives the ball to the owner or drops it within 1.5 m of the owner within 5 seconds after first touching the ball.	frequency
	Gaze alternate	Frequency of gaze alternation (looking from the ball to the owner or from the owner to the ball) within 15 s after the ball has been placed on the window sill by the owner.	frequency
