# Supplementary material for: Personality traits in companion dogs—Results from the VIDOPET
Source: PLoS One. 2018 Apr 10;13(4):e0195448. doi: 10.1371/journal.pone.0195448 (PMC5892901; doi:10.1371/journal.pone.0195448)
Supplement: S2 Table — The variables were rated on a Likert scale from 1–5 (“disagree strongly”, “tend to disagree”, “partly-partly”, “tend to agree”, “agree strongly”). E: experimenter, O: owner. (PDF) [file pone.0195448.s002.pdf]

S2 Table. Variables used in the video rating. The variables were rated on a Likert scale from 1 - 5 (“disagree strongly”, “tend to disagree”, “partly-partly”, “tend to agree”, “agree strongly”). E: experimenter, O: owner.

Subtest	Variable	Further explanation of variable
Exploration	Dependent	spends a lot of time near owner, takes up eye contact
	Active	
	Interested in surroundings	
	Aroused, excited	
Picture	Dependent	spends a lot of time near owner, takes up eye contact
	Active	
	Confident	
	Interested in surroundings	
	Relaxed	
	Aroused, excited	
Greeting the experimenter	Relaxed	
	Aroused, excited	
	Interested in E	
	Passive	
	Greeting intensity	
Food choice	no variables coded	
Focus & Frustration test	Active	
	Focused	is focussed on the sausage or the experimenter
	Motivated	tries many things to obtain the sausage
	Relaxed	
	Aroused, excited	
	Frustrated	e.g. barking, whining, stress signals
Separation	Active	
	Relaxed	
	Aroused, excited	
	Stressed, frustrated	e.g. barking, whining, stress signals, scratching door
	Focused on door(s)	
Greeting after separation	Relaxed E	
	Aroused, excited E	
	Interested in E	
	Passive E	
	Greeting intensity E	
	Relaxed O	
	Aroused, excited O	
	Interested in O	
	Passive O	
	Greeting intensity O	
	Appease O	
Problem solving I (cage)	Active	
	Intensity of efforts	
	Focused on the sausage	
	Stressed, frustrated	e.g. barking, whining, stress signals
	Asks for help	
T-shirt	Relaxed	

Subtest	Variable	Further explanation of variable
	Passive	
	Insecure	
	Stressed	
Obedience	Sit	
	Lay down	
	Come	
	Stay	
	Distractible	
Threatening approach	Confident	
	Appease	
	Alert	how quickly dog shows any behavioural reaction to the threat
	Watchful	aims to maintain its individual distance, direct eye contact with experimenter, ears forward; high arousal level
	Offensive approach	approaches in an agonistic way
	Defensive	shows agonistic behaviour but from a distance
	Avoidance behaviour	
Post-threat interaction	Friendly	shows affiliative behaviour towards E
	Relaxed	
	Aroused, excited	
	Interested in E	
	Passive	
Problem solving II (Bin)	Problem solving ability	
	Asks for help	
Novel object	Active	
	Confident	
	Interested in object	
	Insecure	
	Dependent	
	Careful	
Ball play	Ball motivated	
	Cooperation	interest in playing with O
	Inviting to play	
	Aroused, excited	
	Playfulness	
