# Supplementary material for: Personality traits in companion dogs—Results from the VIDOPET
Source: PLoS One. 2018 Apr 10;13(4):e0195448. doi: 10.1371/journal.pone.0195448 (PMC5892901; doi:10.1371/journal.pone.0195448)
Supplement: S3 Table — The questions were rated on a Likert scale from 1–5 (“disagree strongly”, “tend to disagree”, “partly-partly”, “tend to agree”, “agree strongly”), items marked with an asterisk were reverse coded. The internal consistency of factors calculated from the data of the present study are also shown. (PDF) [file pone.0195448.s003.pdf]

S3 Table. The questionnaire used in the study (DPQ short form, Jones, 2008). The questions were rated on a Likert scale from 1 - 5 (“disagree strongly”, “tend to disagree”, “partly-partly”, “tend to agree”, “agree strongly”), items marked with an asterisk were reverse coded. The internal consistency of factors calculated from the data of the present study are also shown.

**Factor 1 – Fearfulness**		**Cronbach's alpha**: **0.793**
**Facet 1 – Fear of People**		
1*	Dog is relaxed when greeting people.	
6	Dog is shy.	
27	Dog behaves fearfully towards unfamiliar people.	
**Facet 2 – Nonsocial Fear**		
3	Dog is anxious.	
11*	Dog is confident.	
22*	Dog adapts easily to new situations and environments.	
**Facet 3 – Fear of Dogs**		
13	Dog avoids other dogs.	
21	Dog behaves submissively (e.g., rolls over, avoids eye contact, licks lips) when greeting other dogs.	
42	Dog behaves fearfully towards other dogs.	
**Facet 4 – Fear of Handling**		
16	Dog behaves fearfully during visits to the veterinarian.	
35	Dog exhibits fearful behaviours when restrained.	
44	Dog behaves fearfully when groomed (e.g., nails trimmed, brushed, bathed, ears cleaned).	
**Factor 2 – Aggression towards People**		**Cronbach's alpha**: **0.707**
**Facet 1 – General Aggression**		
7	Dog behaves aggressively towards unfamiliar people.	
18*	Dog is friendly towards unfamiliar people.	
40	Dog shows aggression when nervous or fearful.	
**Facet 2 – Situational Aggression**		
25	Dog behaves aggressively in response to perceived threats from people (e.g., being cornered, having collar reached for).	
30	Dog behaves aggressively during visits to the veterinarian.	
36	Dog aggressively guards coveted items (e.g., stolen item, treats, food bowl).	
**Factor 3 – Activity/Excitability**		**Cronbach's alpha**: **0.683**
**Facet 1 – Excitability**		
15	Dog is boisterous.	
31	Dog seeks constant activity.	
41*	Dog tends to be calm.	
**Facet 2 – Playfulness**		
9*	Dog gets bored in play quickly.	
17	Dog enjoys playing with toys.	
33	Dog retrieves objects (e.g., balls, toys, sticks).	
**Facet 3 – Active Engagement**		
4*	Dog is lethargic	
14	Dog works at tasks (e.g., getting treats out of a Kong, shredding toys) until entirely finished.	
24	Dog is curious.	
**Facet 4 – Companionability**		
20	Dog seeks companionship from people.	
26*	Dog is aloof.	
37	Dog is affectionate.	
**Factor 4 – Responsiveness to Training**		**Cronbach's alpha**: **0.646**

**Facet 1 – Trainability**	
29*	Dog is slow to respond to corrections.
38*	Dog ignores commands.
43	Dog is able to focus on a task in a distracting situation (e.g., loud or busy places, around other dogs).
**Facet 2 – Controllability**	
5	When off leash, dog comes immediately when called.
10*	Dog is quick to sneak out through open doors, gates.
32	Dog leaves food or objects alone when told to do so.
**Factor 5 – Aggression towards Animals**	**Cronbach's alpha: 0.673**
**Facet 1 – Aggression towards Dogs**	
2	Dog behaves aggressively toward dogs.
19*	Dog is playful with other dogs.
34*	Dog is friendly towards other dogs.
**Facet 2 – Prey Drive**	
8	Dog likes to chase squirrels, birds, or other small animals.
23	Dog likes to chase bicycles, joggers, and skateboarders.
39	Dog behaves aggressively towards cats.
**Facet 3 – Dominance over other Dogs**	
12	Dog is dominant over other dogs.
28*	Dog willingly shares toys with other dogs.
45	Dog is assertive or pushy with other dogs (e.g., if in a home with other dogs, when greeting).
