# Supplementary material for: Personality traits in companion dogs—Results from the VIDOPET
Source: PLoS One. 2018 Apr 10;13(4):e0195448. doi: 10.1371/journal.pone.0195448 (PMC5892901; doi:10.1371/journal.pone.0195448)
Supplement: S4 Table — For definition of the variables see S1 Table. E: experimenter, O: owner. Loadings > 0.4 are in bold. (PDF) [file pone.0195448.s004.pdf]

S4 Table. Results of the subtest-level PCA analyses of the video coding. For definition of the variables see S1 Table. E: experimenter, O: owner. Loadings > 0.4 are in bold.

Exploration

Variables	Component
Move	**0.839**
Explore	**0.847**
1m from O	**-0.619**
Look O	**-0.787**
Inactive	**-0.927**
Eigenvalue	3.284
Explained variance (%)	65.686

Picture viewing

Variables	Component1	Component2
Move independently	**0.886**	-0.061
Explore	**0.885**	-0.083
Owner 1m	-0.038	**0.993**
Look/ follow O	**-0.812**	0.120
Inactive	**-0.902**	-0.159
Eigenvalue	3.049	1.029
Explained variance (%)	60.980	20.586

Greeting the experimenter

Variables	Component
Approach E score	**0.724**
Greeting E score	**0.873**
Tail wagging	**0.787**
Eigenvalue	1.905
Explained variance (%)	63.488

Food choice

no PCA

Focus & Frustration test

Variables	Component1	Component2
Oriented to Food	**0.855**	0.198
Look E	**-0.851**	0.050
Snap	0.322	**0.681**
Vocalise/ Stress	-0.121	**0.835**
Eigenvalue	1.695	1.080
Explained variance (%)	42.378	27.003

Separation

Variables	Component1	Component2

Near door	-0.157	**0.910**
Look at door	**-0.578**	**-0.691**
Move	**0.877**	-0.066
Explore	**0.845**	-0.217
Inactive	**-0.962**	0.052
Vocalise/ Stress	-0.152	**0.540**
Eigenvalue	2.802	1.638
Explained variance (%)	46.698	27.307

Greeting after separation

**Variables**	**Component1**	**Component2**
Approach latency E	**-0.673**	-0.196
Approach E score	**0.772**	0.162
Greeting E score	**0.792**	0.194
Tail wagging	**0.728**	0.115
Play Intensity	**0.549**	**-0.452**
Approach latency O	-0.020	**-0.762**
Approach O score	0.106	**0.787**
Greeting O score	0.206	**0.639**
Tail wagging	0.304	**0.483**
Eigenvalue	3.077	1.736
Explained variance (%)	34.185	19.293

Problem solving I (cage)

**Variables**	**Component1**	**Component2**	**Component3**
Success latency	-0.262	**-0.732**	-0.008
Oriented to Cage	**0.825**	**0.400**	-0.010
Look O/ E	**-0.848**	0.023	0.193
Owner 1m	0.092	**-0.803**	0.021
Vocalisation	-0.019	-0.007	**0.957**
Latency to give up	**0.748**	-0.042	**0.413**
Eigenvalue	2.224	1.224	1.054
Explained variance (%)	37.072	20.393	17.568

T-shirt

**Variables**	**Component1**	**Component2**
Inactive	**0.947**	-0.242
Look O	**0.878**	-0.327
Follow O	**-0.941**	-0.300
Move independently	-0.268	**0.955**
Eigenvalue	2.774	1.019
Explained variance (%)	69.349	25.470

Obedience

**Variables**	**Component1**	**Component2**
Mean latency to obey commands	**0.685**	0.074

Distract latency	0.138	**-0.702**
Recall latency	0.185	**0.784**
Mean recall latency	**0.827**	-0.123
Mean recall latency from cage	**0.586**	**0.488**
Eigenvalue	1.755	1.160
Explained variance (%)	35.099	23.194

Threatening approach

**Variables**	**Component1**	**Component2**
Latency to aversive reaction	**-0.619**	**-0.499**
Latency to friendly reaction	0.034	**0.847**
Threat reaction	**0.780**	**0.459**
Bark	**0.906**	0.008
Growl	**0.832**	0.104
Approach friendly	-0.275	**-0.780**
Move off	0.117	**0.623**
Eigenvalue	3.487	1.293
Explained variance (%)	49.819	18.475

Post-threat interaction

**Variables**	**Component**
Approach E score	**0.633**
Interaction E score	**0.877**
Tail wagging	**0.828**
Eigenvalue	1.856
Explained variance (%)	61.877

Problem solving II (Bin)

**Variables**	**Component**
Oriented to bin	**0.968**
Success latency	**-0.968**
Eigenvalue	1.874
Explained variance (%)	93.690

Novel object

**Variables**	**Component1**	**Component2**
Owner 1m	0.017	**0.917**
Toy 1m	0.355	**-0.805**
Look O	**-0.739**	0.074
Look Toy	**0.940**	-0.216
Look E	**-0.713**	0.093
Eigenvalue	2.455	1.161
Explained variance (%)	49.098	23.211

Ball play

Variables	Component1	Component2
Follow ball	**0.611**	0.144
Grab ball	**0.755**	0.128
Return	**0.813**	-0.022
Give out ball	**0.614**	-0.290
Gaze alternate	0.060	**0.942**
Eigenvalue	1.988	1.007
Explained variance (%)	39.757	20.140
