# Supplementary material for: Personality traits in companion dogs—Results from the VIDOPET
Source: PLoS One. 2018 Apr 10;13(4):e0195448. doi: 10.1371/journal.pone.0195448 (PMC5892901; doi:10.1371/journal.pone.0195448)
Supplement: S5 Table — E: experimenter, O: owner. Loadings > 0.4 are in bold. (PDF) [file pone.0195448.s005.pdf]

S5 Table. Results of the subtest-level PCA analyses of the video rating. E: experimenter, O: owner. Loadings > 0.4 are in bold.

Exploration

Variables	Component
Dependent	**-0.800**
Active	**0.892**
Interested in surroundings	**0.911**
Aroused, excited	**0.537**
Eigenvalue	2.553
Explained variance (%)	63.828

Picture viewing

Variables	Component1	Component2
Dependent	**-0.421**	-0.272
Active	**0.905**	0.013
Confident	0.253	**0.735**
Interested in surroundings	**0.845**	0.306
Relaxed	-0.098	**0.882**
Aroused, excited	**0.741**	**-0.414**
Eigenvalue	2.378	1.613
Explained variance (%)	39.637	26.876

Greeting the experimenter

Variables	Component1	Component2
Relaxed	-0.110	**0.932**
Aroused, excited	**0.867**	-0.032
Interested in E	**0.561**	**0.696**
Passive	**-0.888**	-0.029
Greeting intensity	**0.886**	0.202
Eigenvalue	2.805	1.243
Explained variance (%)	56.090	24.853

Food choice

No video rating

Focus & Frustration test

Variables	Component1	Component2
Active	**0.895**	-0.114
Focused	**0.508**	**0.653**
Motivated	**0.909**	-0.068
Relaxed	**-0.519**	**0.753**
Aroused, excited	**0.772**	-0.331
Frustrated	0.258	**-0.754**
Eigenvalue	3.154	1.361
Explained variance (%)	52.565	22.681

Separation

**Variables**	**Component1**	**Component2**
Active	0.312	**0.860**
Relaxed	**-0.930**	-0.006
Aroused, excited	**0.719**	0.358
Stressed, frustrated	**0.899**	-0.119
Focused on door(s)	0.196	**-0.874**
Eigenvalue	2.411	1.561
Explained variance (%)	48.212	31.221

Greeting after separation

**Variables**	**Component1**	**Component2**	**Component3**
Relaxed E	-0.147	**0.551**	**0.615**
Aroused, excited E	0.228	**0.407**	**-0.650**
Interested in E	-0.014	**0.917**	0.081
Passive E	-0.264	**-0.642**	0.334
Greeting intensity E	0.191	**0.868**	-0.120
Relaxed O	**0.451**	-0.084	**0.706**
Aroused, excited O	**0.522**	0.063	**-0.547**
Interested in O	**0.645**	0.123	0.313
Passive O	**-0.831**	-0.118	0.364
Greeting intensity O	**0.869**	0.052	-0.004
Appease O	**-0.540**	-0.085	0.080
Eigenvalue	3.473	2.067	1.760
Explained variance (%)	31.574	18.791	16.004

Problem solving I (cage)

**Variables**	**Component1**	**Component2**
Active	**0.616**	-0.351
Intensity of efforts	**0.875**	0.121
Focused on the sausage	**0.844**	0.223
Stressed, frustrated	0.075	**0.926**
Asks for help	**-0.861**	0.051
Eigenvalue	2.611	1,041
Explained variance (%)	52.222	20.827

T-shirt

**Variables**	**Component1**	**Component2**
Relaxed	**0.869**	0.245
Passive	-0.234	**0.801**
Insecure	**-0.852**	0.195
Stressed	-0.369	**-0.740**
Eigenvalue	1.713	1.246
Explained variance (%)	42.831	31.150

Obedience

**Variables**	**Component1**	**Component2**	**Component3**

Sit	**0.863**	0.294	0.013
Lay down	-0.064	**-0.844**	0.109
Come	0.246	**-0.575**	**-0.599**
Stay	0.086	-0.147	**0.909**
Distractible	**-0.820**	**0.414**	0.013
Eigenvalue	1.650	1.247	1.112
Explained variance (%)	32.991	24.944	22.235

Threatening approach

**Variables**	**Component1**	**Component2**
Confident	**-0.612**	0.202
Appease	0.013	**-0.417**
Alert	**0.738**	0.394
Watchful	**0.441**	**0.821**
Offensive (approaches)	-0.105	**0.640**
Defensive	**0.851**	0.047
Avoidance behaviour	**0.549**	**-0.553**
Eigenvalue	2.231	1.682
Explained variance (%)	31.869	24.032

Post-threat interaction

**Variables**	**Component1**	**Component2**
Friendly	**0.637**	**0.698**
Relaxed	-0.206	**0.897**
Aroused, excited	**0.882**	0.014
Interested in E	**0.611**	**0.693**
Passive	**-0.874**	-0.037
Eigenvalue	2.854	1.283
Explained variance (%)	57.076	25.662

Problem solving II (Bin)

**Variables**	**Component**
Problem solving ability	**0.957**
Asks for help	**-0.957**
Eigenvalue	1.832
Explained variance (%)	91.613

Novel object

**Variables**	**Component1**	**Component2**
Active	**0.744**	-0.067
Confident	0.186	**-0.938**
Interested in object	**0.756**	0.082
Insecure	0.004	**0.957**
Dependent	**-0.822**	0.139
Careful	**-0.532**	0.296
Eigenvalue	2.459	1.575
Explained variance (%)	40.988	26.243

Ball play

Variables	Component1	Component2
Ball motivated	**0.844**	0.310
Cooperation	0.194	**0.800**
Inviting to play	0.025	**0.858**
Aroused, excited	**0.687**	-0.138
Playfulness	**0.876**	0.312
Eigenvalue	2.438	1.140
Explained variance (%)	48.761	22.802
